# Resources of India

**Published:** 1859-01

**Authors:** 


### THE EPITOME OF SANITARY LITERATURE.

Resources of India.

DR. FORBES WATSON is preparing an elaborate work on the value, as food, of the various grains which are produced in our Eastern possessions. He has reprinted the introductory chapter under the title of *Remarks on the Development of the Resources of India.* His object is to direct attention to the study of the natural resources of that country, as a means of increasing the material comfort, and consequently the happiness, of the natives. For such a study, the museum opened at the India House, under the management of the late Dr. Forbes Royle, and now entrusted to Dr. Watson, promises to afford abundant material; but that the utility of such an institution may be greatest, it must, as Dr. Watson observes, be truly industrial. It must, whenever possible, contain specimens both of the manufactures, or of the elaborated substances, along with the raw products which have yielded them,—whether these respectively belong to the mineral or the vegetable kingdoms. From such a collection, properly worked by competent men, much may be learned; and already the India House museum is being turned to practical use by Dr. Watson in his researches on the food-grains and pulses. An extract from his pamphlet will shew the great value of the investigations in which he has been engaged.

"We have already in this country excellent collections of the food-grains of the East; but, as they at present stand, they impart no precise notion of their *real* value; and, in the great majority of instances, simply represent a number of small nodular masses, of different shapes and colours, in so many glass jars, and nothing more. This, however, need no longer be the case. Every such sample in future presented to the public, may be employed as an instrument of instruction: it may indicate not only the place of growth and average price in the Indian market, but also its value as an article of food for man or beast. And in addition to furnishing the means of comparison with substances the value of which is well known, these researches farther fulfil the important function of definitely settling what articles are really nutritive, as well as those which are not so.

"The Indian *cereals*, of which forty samples have been submitted to repeated examination, are ten in number. Of rice and wheat little need be said here. . . . . Amongst the remaining eight grains, at least two, from their nutritive and other qualities, are well worthy of attention. These are, the 'great millet,' or Indian *jowaree*, and the 'spiked millet', or *bajra*. A variety of the former is being now introduced into the south of France: its culture has been also extended in Algiers, the climate of which seems admirably adapted to its growth. This kind affords very large returns, excellent sugar, and other products besides. The *bajra* is also a very prolific and valuable grain: it is a great favourite with the natives. The two together form the staple food of a larger population in India than even rice itself, which is considerably inferior to both, as well in nutritive power as in price. They are, in fact, to a vast population, what oatmeal is to Scotland, and maize-flour to America.

"With regard to the numerous *pulses* of India, a few remarks will suffice. They occupy a most important position in the food resources of the country: they are, in fact, to the Brahmin and to the poor, what beef and other flesh are to us; they supply to the muscles that power which neither rice nor other chiefly amylaceous grains are capable of affording. The popular notion that rice forms the entire bulk of the diet of the inhabitants of the East is most fallacious. The stomach of the poor ryot—large as it is—could not with impunity take in and digest the quantity of rice which would be sufficient, day by day, to supply the wants of his system. The pulses, on the other hand, are, as a rule, too rich in exactly that constituent which is deficient in rice. If we attempt to feed men or animals on either one or the other of these *alone*, for even a short period (with, perhaps, one exception, to be presently mentioned), disease and starvation result. Nature's promptings have, however, led the inhabitants of the East to mix these grains together in the quantities necessary to supply the constitutional or innate requirements of his body. . . . .

"In calculating the importance of pulses as articles of food for either man or beast, there are two points from which they must be viewed. As already mentioned, perhaps the whole tribe are incapable of being employed for any length of time, with advantage, *alone*. They are, in one respect, too rich, and must be brought down to a proper standard. The legume, accordingly, which contains *least* of the usually over-abundant flesh-forming substance will, by itself, prove the most nutritious; whereas, on the other hand, the one that contains *most* of the same ingredient will go farthest in yielding to rice or other over-starchy grains the muscle-power in which they are defective. Considered, then, with reference to their nutritive powers, our investigations bring two pulses prominently into relief, as likely to prove of great importance, especially to the agriculturist in this country; and, with regard to one of these, our conclusions are most fully borne out by experience in India and elsewhere. *Gram*, the great horse-food of Western and Northern India, when husked and parched, forms on the voyage, and often on the march, almost the entire food of the Sepoy. In it the essential elements are so fairly balanced, that it can be taken for a longer period than any other of the pea or bean tribe with which we are acquainted; and long employment of it in India has shown that, not only for imparting the requisite strength to animals at work, but also for feeding purposes, it is unrivalled. Throughout the greater part of India it is employed for our horses; and the gram-fed mutton of Bengal has long been famous. Impressed with the importance of its introduction, not only by export from India, but also by actual growth in this country, we have this season succeeded in having the experiment tried on a sufficiently large scale, in different parts of England as well as in Scotland. Failure has occurred in a few instances; but the general result is most favourable. We have already received samples from our friends in the country, the appearance and nutritive qualities of which are found, on examination, to be quite equal to that of the seed originally furnished. Specimens also of the plant have been sent, covered with pods; and we have reason to conclude that the yield is not likely to fall short of, at least, that usually obtained in India.

"In determining the nutritive value of the pulse tribe, we have considered, in the first place, their wholesomeness when eaten *per se;* and in the second, their power of yielding flesh-forming matter to grains poor in that constituent. Hence it is that gram can be employed without injury, for a longer time alone, than any other legume yet known; while others require for their dilution, so to speak, certain admixtures of various kinds. Ignorance, or neglect of this custom, which has been practised in India for ages, has, in Europe, been productive of the most serious consequences. As a proof of this fact, one illustration will suffice. The chickling vetch (*Lathyrus sativus*) contains a larger quantity of nitrogenous or flesh-forming matter than any other of these pulses,—with but one exception, to be presently mentioned. This vetch is still cultivated on the continent; but during the last century it formed, in several parts, a chief aliment of the people, and produced such deleterious effects that the use of it was prohibited by the governments of the countries in which it was raised. When mixed with wheaten flour, or otherwise reduced, it was found to be wholesome; but when employed for some time alone, rigidity of the muscles and paralysis of the limbs resulted. The same effects were also produced on horses and other animals; and Fabbroni, writing from Florence in 1786, states that the government had cautioned the peasants against it as an article of diet, swine having lost the use of their legs from having been exclusively fed on it. Experience, therefore, not only in India, but also in Europe, bears out the conclusion to which, on scientific grounds, we have come.

"The pulse to which we would now call special attention is one calculated to play a most important part as a nutritive substance. *Bhoot* is the native name for it in some parts of India: botanically it is known as *Soja hispida.* It grows not only in India, but also in some of the islands of the Eastern Archipelago, from whence it forms an important article of export to China. Numerous samples of it have been submitted to analysis. It contains not only large proportions of those mineral constituents which are essential to the formation of the frame, but stands unrivalled in its capacity of supplying flesh-forming material to substances deficient in that respect.

"So far chiefly with reference to this country; but, turning to India, the information now afforded of the real nutritive value of the various grains which constitute the staple food of its inhabitants, can be shown to be of still greater importance. As already indicated, experience has taught the native to combine with the grains with which nature has so liberally provided him, certain admixtures which contain the proportions requisite to sustain his bodily strength. A few of these, in every district, are thus employed as articles of diet; but in order to facilitate the practice, and to extend the power of employing *all* the various grain-food products in the same manner, Table No. V. further indicates the proportions in which over-rich pulse grains must be mixed with others in order to produce a wholesome food. Each compound thus artificially formed will contain the essential constituents in the same proportions as in wheat, which we have taken as our standard of comparison; and when dressed in the usual way, with a certain proportion of *ghee*, or fatty matter, will prove equally nutritious. Thus the commissariat officer will be enabled, in accordance with whatever products may for the time be procurable, more readily to furnish a diet calculated to support health and strength; and, in the same manner, in seasons of famine, or during partial failures of the pulse or other crops usually depended on, to point out to those in power the mode of forming various wholesome compounds from other, and for the time, more attainable substances.

"The relative value of the various grains employed in different districts has a very important bearing on a general question affecting the duty of every government. Wherever it is found, whether in Ireland or in India, that the chief article used by the people as food is deficient in nutritive power, every effort should be made to introduce in its stead not one unobjectionable substance, but as many such as practicable. And this remark applies with considerable force to a large tract of country in India, in which the millet now chiefly employed, is, by itself, very innutritious. We allude to a small grain called *raggy* (*Eleusine corocana*), which forms the principal article of diet along the Western Ghauts even in ordinary seasons, and also in many parts of the Deccan. It is very hardy, and extremely prolific; but its very low nutritive power can be readily judged from the large quantity of the various pulses which must be added to it in order to bring it up to the standard. In some parts of the country it is only had recourse to in seasons of scarcity, when it plays the part of a famine food,—and this is its legitimate use; but in those districts, especially, where it is now the grain chiefly depended on, the people should be encouraged, and, if need be, assisted to introduce some of the other millets, the superiority of which can be seen at a glance."

